# A Comparative Analysis of the Outcomes of Various Graft Types in Burn Reconstruction Over the Past 24 Years: A Systematic Review

**DOI:** 10.7759/cureus.54277

**Published:** 2024-02-15

**Authors:** Kenneth Aleman Paredes, Julio C Selaya Rojas, Jose R Flores Valdés, Jaqueline L Castillo, Mauricio Montelongo Quevedo, Francisco J Mijangos Delgado, Hernán A de la Cruz Durán, Carina L Nolasco Mendoza, Edgar J Nuñez Vazquez

**Affiliations:** 1 Surgery, Hospital General Regional No. 220 ¨Jose Vicente Villada¨, Toluca, MEX; 2 Plastic and Reconstructive Surgery, Hospital General Regional No. 220 ¨José Vicente Villada¨, Toluca, MEX; 3 General Medicine, Universidad Autonoma de Guadalajara, Guadalajara, MEX; 4 Surgery, Hospital General Regional No. 220 ¨José Vicente Villada¨, Toluca, MEX; 5 Surgery, Hospital General Regional No. 46, Guadalajara, MEX

**Keywords:** skin burn, autograft harvesting technology, burn reconstruction, plastic and reconstructive surgery, skin graft

## Abstract

Burn injuries, a major global health concern, result in an estimated 180,000 fatalities annually. Despite tremendous progress in treatment methods over the years, the morbidity and mortality associated with burns remain significant. Autologous skin grafting, particularly split-thickness skin grafting (STSG), has been a cornerstone in burn reconstruction, and it has facilitated survival and functional recovery for total body surface area (TBSA) significantly. However, the requirement for primary closure at the donor site due to the constraints of full-thickness donor harvesting continues to pose challenges. The introduction of dermal regenerative templates (DRT) in the late 1970s marked a substantial step forward in tissue engineering, addressing the inadequacy of dermal replacement with STSGs. This systematic review aimed to compare the outcomes of different graft types - bioengineered, autografts, allografts, and xenografts - in burn reconstruction over the last 24 years.

The review focused on the pros and cons of each graft type, offering clinical insights grounded in experience and evidence. The approach involved a systematic review of studies published in English from January 2000 to January 2024, covering randomized controlled trials (RCTs), cohort studies, case-control studies, and case series. The participants comprised individuals of all ages who underwent burn reconstruction with skin grafts, specifically split-thickness grafts, full-thickness grafts, composite grafts, and epidermal grafts (autografts, allografts, and xenografts) and bioengineered grafts. The primary outcomes were functional and cosmetic results, patient satisfaction, graft survival, and complications. The risk of bias was evaluated using the Cochrane risk-of-bias tool for randomized trials version 2 (RoB 2), the Newcastle-Ottawa Scale (NOS) for non-randomized studies, and the Canada Institute for Health Economics (IHE) quality appraisal tool for case series.

Our initial search yielded a total of 1,995 articles, out of which 10 studies were selected for final analysis. Among the four clinical trials assessed, 75% showed a high risk of bias. The studies reviewed involved various graft types, with six studies (60%) concentrating on allografts, three (30%) on autografts, and one (10%) on bioengineered skin grafts. The outcomes were varied, underlining the intricate nature of burn wound management. Our evaluation revealed promising results for autologous-engineered skin substitutes and allografts but also highlighted methodological disparities among the studies included. The dominance of observational studies and the diversity of outcome measures present obstacles to direct comparisons. Future research should address these limitations, employing well-structured RCTs, standardized outcome measures, and exploring long-term outcomes and patient-specific factors. The rapidly evolving field of regenerative medicine offers great potential for novel grafting methods.

This systematic review provides valuable insights into the diverse outcomes of burn reconstruction using different graft types. Autologous-engineered skin substitutes and allografts seem to hold significant promise, suggesting a possible shift in grafting techniques. However, methodological inconsistencies and the lack of high-quality evidence underscore the necessity for further research to fine-tune burn care approaches.

## Introduction and background

Burn injuries are a significant health concern worldwide, often leading to substantial morbidity and mortality; an estimated 180,000 deaths are caused by burns every year [1-3]. In 2008, over 410,000 burn injuries occurred in the United States of America, with approximately 40,000 requiring hospitalizations [3]. Over time, the treatment of these injuries has witnessed significant improvement, mainly due to the development of various grafts. The current gold standard for treating major burns is autologous skin grafting, enabling survival and functional restoration in burn injuries by covering increasingly larger total body surface areas (TBSA) [1].

Several types of skin grafts are used in medical procedures. Split-thickness grafts involve removing the top layer of the skin (epidermis) and a portion of the deeper layer (dermis). These grafts are typically harvested from the front or outer thigh, abdomen, buttocks, or back [4]. Full-thickness grafts remove the epidermis and dermis completely from the donor site, typically the abdomen, groin, forearm, or area above the collarbone. These grafts are often used for minor, highly visible wounds, such as those on the face [4]. Composite grafts contain skin and other tissues, such as cartilage or blood vessels, and are often used when the area to be covered needs more than just skin. Epidermal grafts, which only involve the epidermis, are less common than split-thickness and full-thickness grafts [5]. Grafts can also be classified based on their origin. Autografts are taken from the patient’s own body. Allografts are taken from another person. Xenografts are taken from another species, typically pigs [6]. Synthetic skin substitutes constitute manufactured products that function as skin equivalents [6].

Autologous skin grafting has been a lifesaver in the field of burn care [1]. However, the objective of replacing damaged skin with similar skin structures is often unachievable since full-thickness donor harvesting necessitates primary closure at the donor site for healing. In contrast, split-thickness skin grafting (STSG) only takes a portion of the dermis from the donor site, allowing it to re-epithelialize independently; patients with large defects or more surface to cover will require an allograft [1]. Consequently, STSG is now the main method of wound closure for major burns globally [1].

The creation of the first dermal regenerative template (DRT) in the late 1970s marked a significant breakthrough in tissue engineering. It tackles the problem of inadequate dermal replacement when STSGs are used on full-thickness defects [1]. Despite its advantages, the use of flaps is often restricted in burn patients due to various reasons [2]. This review will offer a comparative analysis of the outcomes of different graft types (autografts, allografts, and xenografts) in burn reconstruction, highlighting the primary advantages and drawbacks of each product and providing practical insights based on clinical experience and evidence. The goal of this systematic review is to compare the outcomes of different graft types in burn reconstruction over time.

## Review

Methods

The current study adhered to the 2020 Preferred Reporting Items for Systematic Reviews and Meta-Analyses (PRISMA) guidelines and employed research grounded in evidence to carry out an exhaustive systematic review [7,8].

Search Strategy

Criteria for inclusion and exclusion were employed to carefully select only studies of high caliber for our analysis. We applied a stringent exclusion criterion to guarantee that the studies incorporated in our analysis were not only of high quality but also pertinent to our research objectives. On 01/04/2024, we initiated a comprehensive search across three major databases: PubMed (Table 1), ScienceDirect (Table 2), and Cochrane (Table 3). Our search strategy involved the use of Medical Subject Heading (MeSH) terms, Boolean operators, and free text terms to ensure a wide coverage of relevant studies.

**Table 1:** PubMed search Specific search string used to identify relevant articles on PubMed/Medline database. Search and extraction date: 01/04/2023 MeSH: Medical Subject Heading

Search terms	Results
("Skin Transplantation"[MeSH Terms] OR "skin, artificial"[MeSH Terms] OR "Transplants"[MeSH Terms] OR "Graft Survival"[MeSH Terms]) AND "Burns"[MeSH Terms] AND ("Treatment Outcome"[MeSH Terms] OR "Patient Outcome Assessment"[MeSH Terms])	781

**Table 2:** ScienceDirect search Specific search string used to identify relevant articles on the ScienceDirect database. Search and extraction date: 01/04/2023

Search terms	Results
Burn patients' reconstruction treatment AND graft transplant	1,542

**Table 3:** Cochrane search Specific search string used to identify relevant articles on Cochrane Library. Search and extraction date: 01/04/2024

ID	Search terms	Results
#1	Burn reconstruction AND graft types	8
#2	“"burn reconstruction"”	89
#3	(Graft types):ti,ab,kw	680
#4	(Burn patients):ti,ab,kw	3,607
#5	(Treatment for burn reconstruction)	53
#6	(burn treatment):ti,ab,kw	2,900
#7	Skin grafts	705
#8	Autologous graft	2,084
#9	(allografts AND reconstruction	102
#10	Xenografts AND burn patients	3
#11	#1 OR #2 OR #3 OR #4	149
#12	#5 OR #6 OR #7 OR #8 OR #9 OR #10	66
#13	(#11 OR #12) AND (#5 OR #9)	215

*Inclusion and Exclusion Criteria* 

Types of study*: *We searched for pertinent studies published in English from January 2000 to January 2024. We thoroughly examined randomized controlled trials (RCTs), cohort studies, case-control studies, and case series. We ruled out systematic reviews, meta-analyses, narrative reviews, and most of the studies that are observational or at the bottom of the hierarchy of scientific evidence. Additionally, we discarded studies that failed to provide a lucid description of their procedures, duplicates, and those from which we could not procure the necessary data, those complete texts we could not view, or those from whose original author we did not receive a response via email.

Types of participants*: *Our study established specific selection criteria for participants, which were as follows: articles with patients of any age who had undergone burn reconstruction in the form of a skin graft, including articles that only report the type of skin graft, such as STSG, full-thickness grafts, composite grafts, and epidermal grafts (autografts, allografts, and xenografts), and bioengineered graft. We excluded studies involving any type of treatment other than a skin graft, studies involving animals or in vitro models, studies involving patients who had not undergone burn reconstruction, or those where the burn reconstruction did not involve grafting. We aimed to include a diverse range of participants to gain a more comprehensive understanding of the intervention.

Types of intervention: Interventions involving any type of graft used in burn reconstruction such as autologous skin grafting, DRT, perforator-based interposition flaps, full-thickness skin grafts, and bioengineered grafts from any origin, such as autologous, xenograft, and allografts. The control group could involve those without any intervention, those involving standard care, or alternative intervention studies that did not involve a graft or where the type of graft used is not clearly specified.

Outcomes: The outcome measures included studies that reported on the outcomes of burn reconstruction with different graft types; this could include functional outcomes, cosmetic outcomes, patient satisfaction, graft survival, and complications. We excluded studies that did not report outcomes or where the outcomes were not clearly defined.

Data Extraction

Selection of studies:* *After an initial review based on the titles and abstracts, two reviewers (JLC, KAP) independently chose trials to be included in this review, guided by pre-established inclusion and exclusion criteria. The Rayyan web app [9] was utilized in this search to extract pertinent data, and duplicate entries were removed. Keywords were used to aid with the search [9]. Any disagreements regarding the inclusion of studies were settled through consensus and consultation with a third author (JRFV). For the full-text screening, two reviewers (JLC, KAP) independently picked trials for inclusion, again using predetermined inclusion and exclusion criteria. This search was conducted using Rayyan [9] (to extract relevant data, and duplicates were filtered out; keywords were used) [9]. Disagreements about the inclusion of studies were again resolved through consensus and consultation with a third review author (JRFV).

Data Evaluation

Assessment of risk of bias in included studies: We performed the data evaluation by using the standards outlined in Cochrane. To gauge the quality of the studies included, we utilized the Cochrane RoB 2.0 tool [10], which scrutinizes potential bias in RCTs. For case-control and cohort studies, we applied the Newcastle-Ottawa Scale (NOS) [11]. Finally, for case series, we used the Canada Institute for Health and Economics (IHE) quality appraisal tool [12].

Two independent reviewers (FJMD, MMQ) assessed the risk of bias in each study, taking into account the specific criteria and guidelines provided by the respective tools. Any disagreements between the reviewers were resolved through discussion or, if necessary, by consulting with a third, blinded reviewer. The methodological components of the trials and case-control, cohorts and case series were "a low, high, or unclear risk of bias" according to the Cochrane Handbook for Systematic Reviews of Interventions, NOS, and IHE guidelines, respectively [11-13]. 

Results

**Figure 1:**
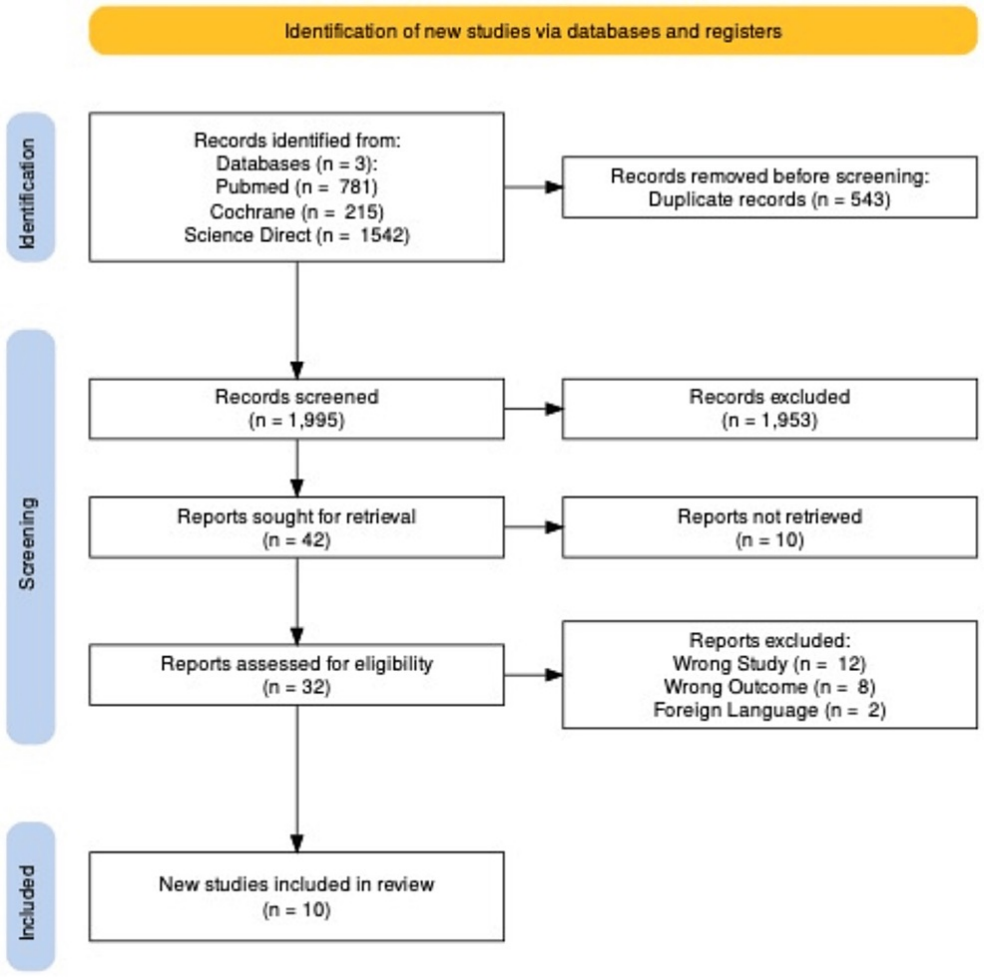
PRISMA flow chart The flow diagram, which is based on the PRISMA statement [7], outlines our process. We initially identified 1,995 articles from three separate databases. Out of these, 42 were retrieved, and 32 were evaluated for their suitability. Finally, we included 10 articles in this systematic review after a thorough assessment PRISMA: Preferred Reporting Items for Systematic Reviews and Meta-Analyses

The process of identifying and selecting studies from the database resulted in a shortlist of 1,995 articles. Upon careful scrutiny, 543 duplicate articles were removed. The initial screening of titles and abstracts led to the selection of 32 publications for a more detailed review, which involved accessing the full texts. After assessing the suitability and quality of these shortlisted full-text papers, 10 articles were chosen for the final review process. The procedure for selecting the studies is illustrated in the PRISMA flow chart in Figure 1.

**Figure 2:**
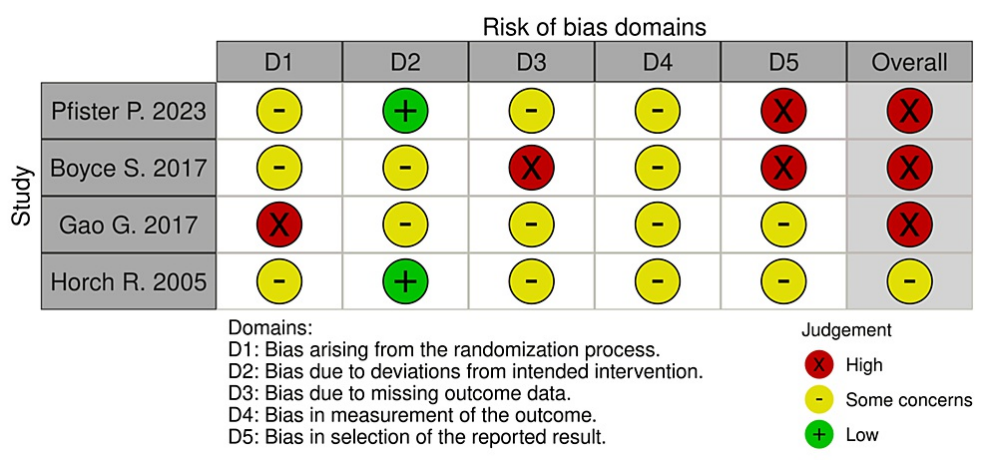
Risk of bias assessment for randomized controlled studies The evaluation was carried out using the Cochrane risk-of-bias tool for randomized trials [10]. Each article was assessed for bias risk. Of the four articles evaluated, three exhibited a high risk of overall bias [14-16]. In comparison, one article showed some concern for bias [17]

**Figure 3:**
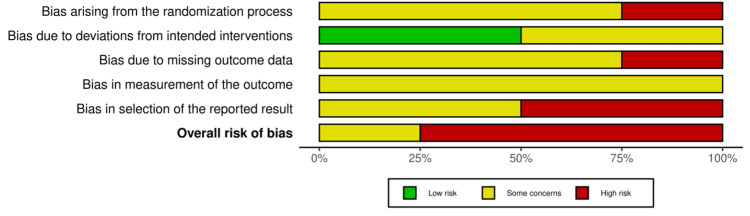
Traffic light plot for randomized controlled trials This figure shows part of the risk of bias, and the overall results are depicted with three colors: green (low risk), yellow (some concerns), and red (high risk) [10]

Of the four clinical trials evaluated, 0 (0%) showed an overall low risk of bias, one (25%) showed some concern for bias, and three (75%) had a high risk for bias. Of the one study evaluated using NOS, one (100%) showed good quality (Figures 2-3, Table 4). The appraisal for the case reports and case series is depicted in Table 5.

**Table 4:** Risk of bias assessment with the Newcastle-Ottawa Scale for non-randomized studies Good quality: 3 or 4 stars in the selection domain AND 1 or 2 stars in the comparability domain AND 2 or 3 stars in the outcome/exposure domain. Fair quality: 2 stars in the selection domain AND 1 or 2 stars in the comparability domain AND 2 or 3 stars in the outcome/exposure domain. Poor quality: 0 or 1 star in the selection domain OR 0 stars in the comparability domain OR 0 or 1 star in the outcome/exposure domain

Study	Study design	Selection	Comparability	Outcome/exposure	Total	Subjective evaluation
Chua et al., 2004 [18]	Cohort	4	1	3	8	Good quality
Chan et al., 2013 [19]	Cohort	2	1	3	6	Fair quality

**Table 5:** Risk of bias assessment for case reports and case series This table presents a risk of bias assessment for case reports and case series from four different studies cited above. We employed the appraisal tool by The Canada Institute of Health Economics (IHE) [12]. The assessment was based on a set of leading explanatory questions across various domains such as "study objective, study design, study population, intervention and co-intervention, outcomes measures, statistical analysis, results and conclusions, and competing interests and sources of support". The responses to these questions were categorized as "Yes," "No," or "Unclear" for each study

Domain	Leading explanatory question	Moravvej et al., 2012 [20]	Coruh and Yontar, 2012 [21]	Zidan and Eleowa, 2013 [22]	Jiaqi et al., 2005 [23]
Study objective	1. Was the hypothesis/aim/objective of the study clearly stated?	Yes	Yes	Yes	Yes
Study design	2. Was the study conducted prospectively?	Yes	Unclear	Yes	Unclear
	3. Were the cases collected from more than one center?	No	No	No	No
	4. Were patients recruited consecutively?	Yes	Unclear	Unclear	Yes
Study population	5. Were the characteristics of the patients included in the study described?	Yes	Yes	Yes	Yes
	6. Were the eligibility criteria (i.e., inclusion and exclusion criteria) for entry into the study clearly stated?	Yes	Yes	Yes	Yes
	7. Did patients enter the study at a similar point in the disease?	Unclear	Unclear	Unclear	Unclear
Intervention and co-intervention	8. Was the intervention of interest clearly described?	Yes	Yes	Yes	Yes
	9. Were additional interventions (co-interventions) clearly described?	Yes	Yes	Yes	Unclear
Outcomes measures	10. Were relevant outcome measures established a priori?	Unclear	Unclear	Unclear	Unclear
	11. Were outcome assessors blinded to the intervention that patients received?	Unclear	Unclear	Unclear	Unclear
	12. Were the relevant outcomes measured using appropriate objective/subjective methods?	Unclear	Yes	Unclear	Unclear
	13. Were the relevant outcome measures made before and after the intervention?	Unclear	Yes	Unclear	Unclear
Statistical analysis	14. Were the statistical tests used to assess the relevant outcomes appropriate?	Unclear	Unclear	Unclear	Unclear
Results and conclusions	15. Was follow-up long enough for important events and outcomes to occur?	Yes	Yes	Yes	Yes
	16. Were losses to follow-up reported?	Unclear	Unclear	Unclear	Unclear
	17. Did the study provide estimates of random variability in the data analysis of relevant outcomes?	Unclear	Unclear	Unclear	Unclear
	18. Were the adverse events reported?	Yes	Unclear	Yes	Yes
	19. Were the conclusions of the study supported by the results?	Yes	Yes	Yes	Yes
Competing interests and sources of support	20. Were both competing interests and sources of support for the study reported?	Yes	Unclear	Unclear	Unclear

Of the 10 studies examined, six (60%) used allografts or allogenic skin grafts in the burn reconstruction process, three used autograft skin grafts (30%), and only one used the bioengineered skin graft (10%). These studies explored various factors such as the degree of burn, sample size, intervention methods (such as allogenic, autograft, and bioengineered), controls, and reconstruction techniques (such as split-thickness skin graft and dermal graft). The goal was to gain valuable insights into burn wound management by assessing aspects like scar flexibility, blood supply, and sensory outcomes. The results highlighted that treating burns involves many different aspects.

For example, Pfister et al. [14] conducted an RCT involving 30 participants, focusing on allogenic and autologous skin grafts using the STSG technique. The research indicated the potential advantages of autologous-engineered skin substitutes over split-thickness skin autografts, such as the reduced need for donor skin and lower mortality rates. Conversely, Moravvej et al.'s case series [20] of 14 patients with third-degree burns showed faster healing and less scar formation with alloskin grafting compared to petroleum jelly-impregnated gauze. These results collectively highlight the variety of methods used in burn treatment and underscore the necessity for additional research and clinical trials to determine the efficacy and feasibility of different graft types and techniques. The studies acknowledged their limitations such as those related to sample size, absence of randomization, and the requirement for longer follow-up periods, singling out areas for future exploration in burn care. More results are shown in Table 6.

**Table 6:** General outcomes of included studies RCT: randomized controlled trial; N/A: not applicable; AMs: amniotic membranes; FTSG: full-thickness skin graft; SSG: split-skin graft; STSG: split-thickness skin graft; ESS: engineered skin substitute; TBSA: total body surface area; POD: postoperative day; STDG: split-thickness dermal graft; GPA: glycerol-preserved allograft

Study	Study design	Burn degree	Sample size	Intervention (type of graft)	Controls	Reconstruction method	Result	Comment
Pfister et al., 2023 [14]	RCT	Superficial partial thickness, deep partial thickness, or full thickness burn	30	Allogenic	Autologous skin allograft	STSG	N/A	This study could contribute valuable insights to the field of burn wound management and might pave the way for more widespread use of biological dressings like AMs in clinical practice. However, the results of the study would need to be evaluated upon completion to determine the effectiveness and practicality of this approach
Chan et al., 2013 [19]	Cross-sectional	NA	26	FTSG and STSG	At a mean of 13.5 months post-surgery, pliability was significantly enhanced in FTSG compared with SSG (p<0.001). Vascularity and height of SSGs were preferred, although not statistically significant. Sensation did not differ significantly between the two types of grafts	12 FTSGs and 10 STSGs	At a mean of 13.5 months post-surgery, pliability was significantly enhanced in FTSG compared with SSG. Vascularity and height of SSGs were preferred, though not statistically significant. No difference in sensation between the two types of graft or donor site outcomes. Hair growth was significantly more prominent in FTSG. Equal number of contractures in both groups, with grafts involving both palms and digits more commonly affected	The study indicates that FTSG can lead to improved outcomes, especially in scar pliability in children with deep palm burns. However, hair growth is a cosmetic concern associated with FTSG. The study acknowledges its limitations, including the lack of randomization, small sample size, and the need for more long-term follow-up. To address these limitations, the study suggests conducting a multicenter, prospective, RCT for a more comprehensive analysis
Moravvej et al., 2012 [20]	Case- series	Third-degree burns	14	Allografts	Petroleum jelly-impregnated gauze	Meshed STSG	The study found that alloskin had better properties than petroleum jelly-impregnated gauze for burn treatment. The healing time was significantly shorter in the alloskin group (8.8 days) compared to the petroleum jelly group (13.6 days). Alloskin also resulted in less scar formation and a pigmentation score closer to normal. However, the difference in scar formation became insignificant after 12 months, and no differences were observed between the two groups after a one-year follow-up. This suggests that the long-term outcomes of both treatments may be similar	The study found that alloskin grafting, which uses allofibroblasts on meshed STSG, can be beneficial for burn healing. It led to faster healing, less scar formation, and reduced pigmentation compared to the traditional method using petroleum jelly-impregnated gauze. However, the improvements in scar formation were not sustained after a year, indicating that the long-term results might be similar for both methods. The study emphasizes the potential advantages of using alloskin in the initial stages of burn treatment
Boyce et al., 2017 [15]	RCT	Full-thickness burns, 76.9% TBSA	16	Bioengineered	Autograft	Autologous ESS and split-thickness skin autografts (AG)	Autologous ESS significantly reduced the need for donor skin compared to split-thickness skin AG, with a ratio of closed wound area to the donor skin area of 108.7 for ESS vs. a maximum of 4.0 for AG. The mortality rate for subjects was 6.25%, which is significantly lower than a comparable population from the National Burn Repository (NBR). Engraftment was slightly lower for ESS (83.5%) compared to AG (96.5%). The percentage of TBSA closed at POD 28 was 29.9% for ESS and 47.0% for AG, showing significant differences between the graft types. A positive correlation (R2=0.65) was found between the percentage of TBSA closed with ESS and the percentage of TBSA full-thickness burn. The reduction in donor skin requirements for ESS suggests potential benefits in terms of reduced morbidity and improved long-term recovery	The study used statistical analyses, including t-tests, Fisher’s exact test, and correlation analyses, to compare outcomes between ESS and AG. The results indicate a potential medical benefit of autologous ESS in the treatment of extensive, full-thickness burns
Coruh and Yontar, 2012 [21]	Case series	Deep partial and full-thickness burn	9	Allograft or autograft	N/A	STDG	The study applied STDGs to 11 patients with deep partial- and full-thickness burns over a year. The graft was successful in all but one patient, with no significant issues reported at the donor site. The healing of the donor site was not problematic, although epithelization generally took a week longer than with STSGs. The study suggests that STDGs could be a new method for auto-skin grafting in extensive deep partial- and full-thickness burns	The authors suggest that STDGs could be a beneficial supplement to auto-skin grafting techniques, especially in situations where autologous STSG might be restricted. They introduce a new method of using STDGs from the same donor site during the same surgical procedure, which could potentially offer more autologous skin graft material
Gao et al., 2017 [16]	RCT	Third-degree burns (full-thickness burns)	105	Autografts	N/A	STSG (Meek, Stamp, and Microskin)	The skin graft survival rate was significantly higher in the Meek group compared to the rates in the Stamp and Microskin groups (both p<0.01). In addition, the skin graft fusion time, wound healing time, and 1% TBSA treatment costs were significantly lower in the Meek group compared to those in the Stamp and Microskin groups (both p<0.01). Furthermore, the Meek group exhibited better results for curative efficacy, scarring status, and joint activity in comparison to the other two groups (both p<0.05)	The Meek skin graft method is superior to the Stamp and Microskin methods in several aspects, including survival rates, fusion time, wound healing, treatment costs, overall efficacy, and scarring. It is particularly effective and cost-efficient for treating third-degree burns
Zidan and Eleowa, 2013 [22]	Case series	Not specified	24	Allograft	N/A	FTSG	The study focused on the use of GPA in burn and wound management. Over a year, 22,000 cm² of skin was gained from body contouring procedures. This harvested GPA was utilized in 28 surgeries for 24 patients. The graft success rate was high, with only three cases (12.5%) experiencing partial graft loss and one case (4%) experiencing total graft loss. A majority of patients (87.7%) reported excellent graft take, where the grafts adhered well to the underlying bed with no significant separation or collection underneath. The study underscored the effectiveness of GPA in burn and wound management, particularly in situations where the use of cadaveric skin is not legalized	The study suggests that skin grafts preserved with glycerol and obtained from body contouring procedures could be a viable alternative for treating burns and wounds, especially in areas where the use of cadaveric skin is not legal. It encourages more research and application of this method to overcome the issue of skin graft scarcity in these areas. The results show promising outcomes, including good graft adherence and potential advantages in managing chronic and complex wounds
Jiaqi et al., 2005 [23]	Case series	Eyelids burn	15	Allograft	N/A	Acellular dermal allograft	All patients achieved satisfactory function and appearance, with no instances of implant rejection or severe complications reported. One specific case highlighted involves a patient with chemical burns who showed symptom resolution after six months	The study suggests that acellular dermal allograft may be a safe and effective option for posterior lamellar spacer graft in eyelid reconstruction after chemical and thermal burns. The lack of rejection and minimal inflammation observed in the study supports the biocompatibility of acellular dermal allograft. However, the study is limited by its small sample size, absence of a control group, and relatively short follow-up period. Further research with larger sample sizes and comparative studies with different graft types could provide more robust evidence for the efficacy of acellular dermal allograft in eyelid reconstruction
Horch et al., 2005 [17]	RCT	Superficial and deep partial thickness facial burn	10	Allograft + silversulfadiazine ointment	Allograft + early superficial debridement	Cadaveric allograft	Time to reepithelialization was significantly faster in the glycerolized cadaver skin group (10.5 days) compared to the silversulfadiazine group (12.4 days) (p<0.05). Scar quality was significantly improved in the glycerolized cadaver skin group. At three and six months post-burn, no patients had significant hypertrophic scarring in the glycerolized cadaver skin group, while two patients in the silversulfadiazine group developed hypertrophic scars (p<0.05)	The study found that using glycerolized cadaver allograft skin for treating facial burns led to better results in terms of faster reepithelialization and improved scar quality compared to the standard treatment with silversulfadiazine. It suggests that this type of allograft skin could be a useful biological dressing for both shallow and deep partial-thickness facial burns. However, the study recommends conducting more clinical studies with a larger patient population to further assess the effectiveness of allogenic skin for facial burns
Chua et al., 2004 [18]	Cohort study	Deep dermal to full-thickness burns, 30% TBSA	45	Autograft/allograft	Debridement	Skin autograft/allograft transplantation against those who received conventional staged excision and coverage	Patients with early excision had surgery 1.7 days post-injury on average, compared to 5.5 days for the control group. The mortality rate decreased significantly from 45% in the control group to 16% in the study group. The length of hospital stay decreased significantly from an average of 58.5 days in the control group to 48.3 days in the study group	The study indicates that early complete excision with skin autograft/allograft transplantation can significantly reduce the mortality rate and hospital stay length for severe burn patients compared to conventional staged excision and coverage. These findings, which are supported by previous studies, confirm the effectiveness of early excision and grafting in severe burn management. However, the study acknowledges the limitation of its small sample size and proposes a larger retrospective review in the future. The study also emphasizes the importance of the skin bank in Singapore, which provides a readily available source of skin allograft for burn victims

These findings led us to conclude that the best practice for burn reconstruction is to promptly remove the damaged tissue and close the wound using one of the traditional methods, such as autograft.

Discussion

This systematic review shed light on the varied outcomes of burn reconstruction with different graft types. Despite methodological differences across the 10 studies, certain palpable trends emerged. Autologous-engineered skin substitutes showed potential benefits over traditional autografts, including a reduced need for donor skin and lower mortality rates. Allografts, especially alloskin grafting, offered faster healing and less scarring compared to petroleum jelly-impregnated gauze. Bioengineered skin grafts, though limited to one study, suggested less morbidity and better long-term recovery for extensive, full-thickness burns.

These findings echo previous research supporting the superiority of autologous grafts in certain situations. The potential advantages of autologous engineered skin substitutes could lead to a shift in grafting techniques, thereby reducing donor site morbidity and improving patient outcomes [15]. Allografts, particularly alloskin, could provide a valuable alternative for burn treatment, underscoring the need to explore different graft types based on patient characteristics and burn severity. The results also highlight the importance of innovative approaches, like bioengineered skin substitutes, in enhancing burn care outcomes.

This review has a few limitations, including the heterogeneity of study designs, patient populations, and outcome measures, making direct comparisons challenging. The predominance of observational studies and the lack of high-quality RCTs could introduce biases. The evaluated studies showed varying bias risks, emphasizing the need for further high-quality research to strengthen the evidence base in the field of burn reconstruction. Future research should address these limitations and enhance our understanding of burn reconstruction outcomes. We recommend further well-designed, multicenter, RCTs comparing different graft types, employing standardized outcome measures, and addressing patient population variability. Exploring the long-term outcomes of different graft types and investigating the impact of patient-specific factors on graft success would help refine burn care strategies. Future research should focus on developing novel grafting techniques and materials, considering the evolving field of regenerative medicine. Bioengineered skin substitutes, as demonstrated in the study by Boyce et al., show significant promise.

## Conclusions

The systematic review of 10 studies revealed a diverse range of outcomes in burn reconstruction with various graft types. Autologous engineered skin substitutes and allografts, particularly alloskin grafting, demonstrated great potential for positive outcomes over traditional methods. These findings suggest an emerging shift in grafting techniques that could enhance patient outcomes and reduce donor site morbidity. However, the heterogeneity of study designs and patient populations and the predominance of observational studies underscores the need for further high-quality multicenter RCTs. Future research should focus on comparing different autologous-engineered skin substitutes, exploring long-term outcomes, and investigating the impact of patient-specific factors on graft success. The evolving field of regenerative medicine also presents opportunities for developing novel grafting techniques and materials. While our review provides valuable insights into burn reconstruction outcomes, it also highlights the complexities of this field and the need for further research to refine burn care strategies with techniques such as autologous-engineered skin substitutes.
